# Molecular Detection of Linezolid Resistance Determinants and Identification of a Novel C2626T Mutation in Clinical Isolates of *Enterococcus* Species from India

**DOI:** 10.3390/antibiotics15090841

**Published:** 2026-08-31

**Authors:** Madhumala Shanmugasundaram, MuthuLakshmi BackiaSubramanian, Shanthi Mariappan, Uma Sekar, Kennedy Kumar Palraj, Binesh Lal Yesudhason, Rhea Michelle J. Khodabux

**Affiliations:** 1 Department of Microbiology, Sri Ramachandra Institute of Higher Education and Research, Chennai 600116, Tamil Nadu, India; madhumalas@sriramachandra.edu.in (M.S.); muthulakshmib@sriramachandra.edu.in (M.B.); umasekarsrmc@gmail.com (U.S.); michellekhodabux@gmail.com (R.M.J.K.); 2 Department of Clinical Microbiology, Christian Medical College, Vellore 632004, Tamil Nadu, India

**Keywords:** linezolid resistance, *Enterococcus* species, *cfr(D)* gene, *optrA* gene, 23S rRNA mutation in domain V, healthcare

## Abstract

**Background**: *Enterococcus* species have emerged as significant multidrug-resistant nosocomial pathogens. Linezolid remains a vital last-resort therapeutic option for the management of severe infections caused by vancomycin-resistant *Enterococci*. However, resistance to linezolid occurs through several mechanisms, including the presence of the *cfr*, *cfr(D)*, and *optrA* genes, as well as mutations in domain V region of the 23S ribosomal RNA gene. This study aimed to detect and characterize linezolid resistance in clinical *Enterococcus* species. **Methodology**: A total of 266 clinical isolates belonging to the *Enterococcus* species were analyzed in this study. Antimicrobial susceptibility testing was performed using the disc diffusion method. The Minimum Inhibitory Concentration (MIC) of linezolid was determined by the agar dilution technique. PCR was performed to detect the presence of *cfr*, *cfr(D)*, and *optrA* genes, along with mutations in domain V of the 23S rRNA gene. **Results**: Among the 266 isolates analyzed, 25 (9.4%) were found to be resistant to linezolid, with MIC values ≥ 8 µg/mL. Of these resistant isolates, the *cfr* gene was detected in one isolate, *cfr(D)* in sixteen isolates, and *optrA* in nine isolates. Notably, four isolates carried mutations in the domain V region of the 23S rRNA gene, including a novel C2626T mutation (in India) along with the previously reported G2592T mutation. **Conclusions**: This study reports the detection of *cfr*- and *cfr(D)*-mediated linezolid resistance among *Enterococcus* species in India. Furthermore, the presence of *optrA* and a novel C2626T mutation, alongside the G2592T mutation, was identified among resistant isolates. These findings underscore the urgent need for molecular surveillance to prevent further dissemination of these multidrug-resistant pathogens.

## 1. Introduction

*Enterococci* are Gram-positive cocci and gastrointestinal commensals. They are significant nosocomial pathogens leading to various infections such as urinary tract infections, skin and soft tissue infections, and intra-abdominal infections. Moreover, they are associated with conditions such as endocarditis, bacteremia, and meningitis [1]. *Enterococcus faecalis* and *Enterococcus faecium* are the foremost *Enterococcus* species detected in clinical isolates. Treatment options are dwindling due to their intrinsic resistance to various commonly used antimicrobial agents (particularly cephalosporins and low-level aminoglycosides) and their propensity to quickly acquire resistance [2]. 

Linezolid is a synthetic bacteriostatic antimicrobial within the oxazolidinone class, widely utilized in clinical settings to combat infections acquired in the hospital as well as those acquired within the community because of its high oral bioavailability [3,4]. It exerts its antibacterial activity by binding to a specific site on the 23S ribosomal RNA of the 50S ribosomal subunit, thereby inhibiting bacterial protein synthesis and preventing the formation of the 70S initiation complex. It is considered a last-resort antimicrobial treatment for multidrug-resistant Gram-positive bacteria, notably vancomycin-resistant *Enterococci* [4]. Among *Enterococci*, vancomycin-resistant *Enterococci* (VRE) are a major clinical concern, with reported global mortality rates ranging from 60% to 70% [5]. In 2002, an initial case of linezolid-resistant *Enterococci* was reported from the UK [6]. Subsequently, Kumar et al. (2014) unveiled the initial report of linezolid-resistant *Enterococci* in India [7]. In light of the escalating detection of linezolid-resistant *Enterococci* in recent years, there is an imperative need for extensive research into the mechanisms behind their resistance.

Linezolid resistance in *Enterococcus* is primarily attributed to mutations in the 23S ribosomal RNA, which alter the target site of linezolid and disrupt its binding efficacy [8]. Apart from these mutational mechanisms, highly efficient and easily transmissible non-mutational resistance has also been identified. One such mechanism involves the *cfr* gene, which encodes the *cfr* methyltransferase. This enzyme modifies the adenine at position 2503 in the 23S rRNA, leading to resistance against five distinct classes of antimicrobials: pleuromutilins, streptogramin A, oxazolidinones, phenicols, and lincosamides [9,10]. The presence of the *cfr* gene on mobile genetic elements such as plasmids and transposons facilitates its horizontal transfer among bacterial species and contributes to the dissemination of antimicrobial resistance. A *cfr*-like variant, the *cfr(D)* gene, exhibits a 64 percent similarity in amino acid sequence within the *cfr* gene [11,12]. Another transferable resistance mechanism is associated with the *optrA* gene, which encodes an ATP-binding cassette (ABC-F) protein. This protein mediates resistance to oxazolidinones, such as linezolid and tedizolid, by protecting the bacterial ribosome from the action of these antibiotics [13]. Linezolid-resistant enterococci (LZRE) are most commonly associated with urinary tract and bloodstream infections [14]. Moreover, infections caused by LZRE have been linked to a significantly increased risk of in-hospital mortality compared with linezolid-susceptible enterococcal infections (OR 9.3; 95% CI: 1.8–51.2) [15]. Only a limited number of studies from India have investigated the mechanisms of linezolid resistance in *Enterococcus*. Among these studies, mutations in the 23S rRNA and *optrA*-mediated resistance were most commonly observed [16,17]. This study aimed to characterize the distribution of *optrA*, *cfr*, *cfr(D)*, and 23S rRNA mutations in linezolid-resistant *Enterococcus* isolates.

## 2. Results

**Figure 1:**
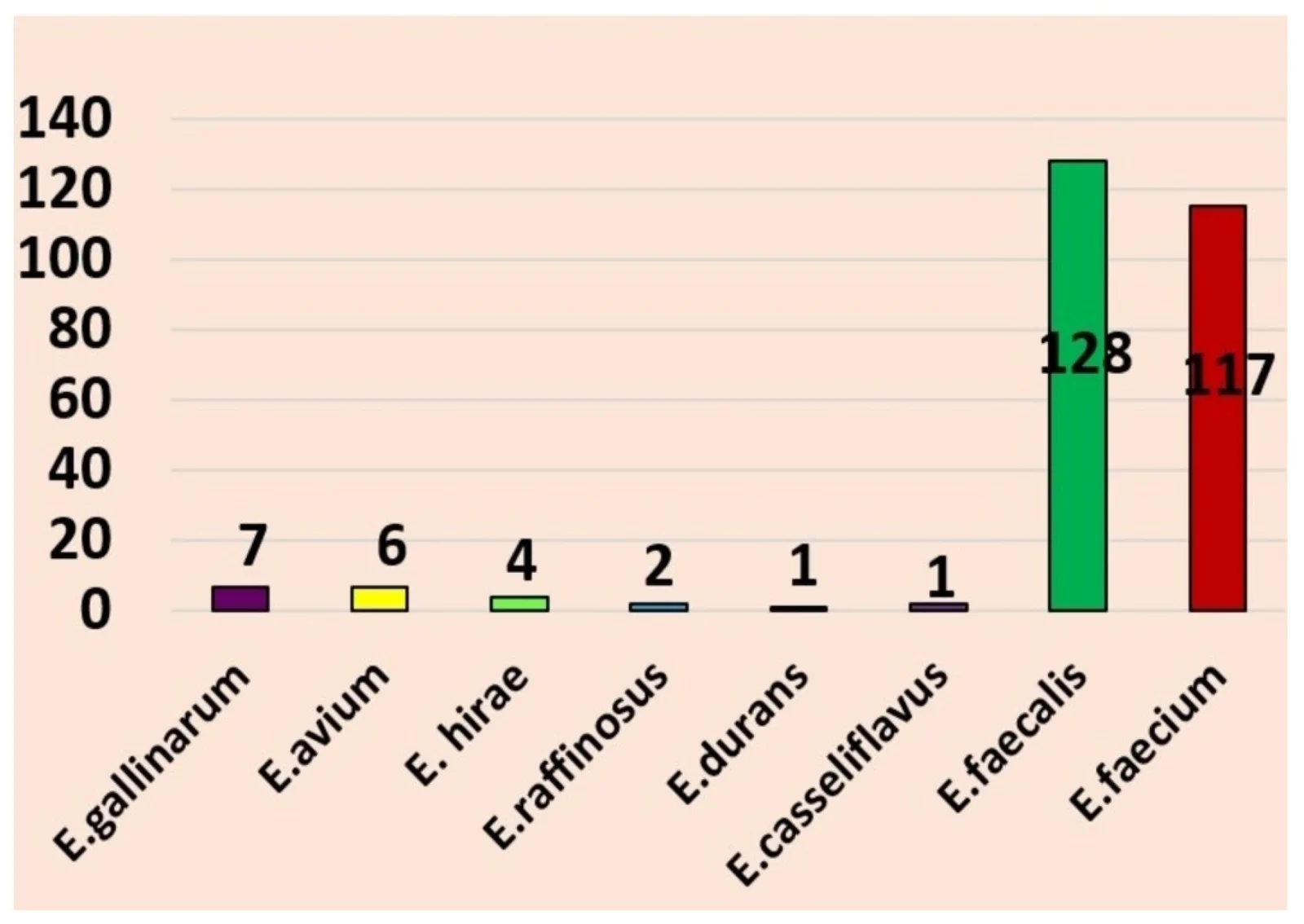
Distribution of *Enterococcus* species among clinical isolates.

A total of 266 isolates were examined in this study. Among them, *Enterococcus faecalis* predominated (128; 48.1%), followed by *E. faecium* (117; 44.0%), *E. gallinarum* (7; 2.6%), *E. avium* (6; 2.3%), *E. hirae* (4; 1.5%), *E. raffinosus* (2; 0.7%), *E. casseliflavus* (1; 0.4%), and *E. durans* (1; 0.4%). The species-wise distribution of *Enterococcus* isolates is shown in Figure 1.

### 2.1. Antimicrobial Susceptibility Pattern

The antimicrobial resistance patterns are listed as follows: ampicillin (57%, 152/266), ciprofloxacin (84.6%, 83/98), nitrofurantoin (52%, 51/98), erythromycin (82.1%, 138/168), vancomycin (27.8%, 74/266), and linezolid (9.4%, 25/266).

### 2.2. Minimum Inhibitory Concentration of Linezolid-Resistant Enterococcus Species

**Figure 2:**
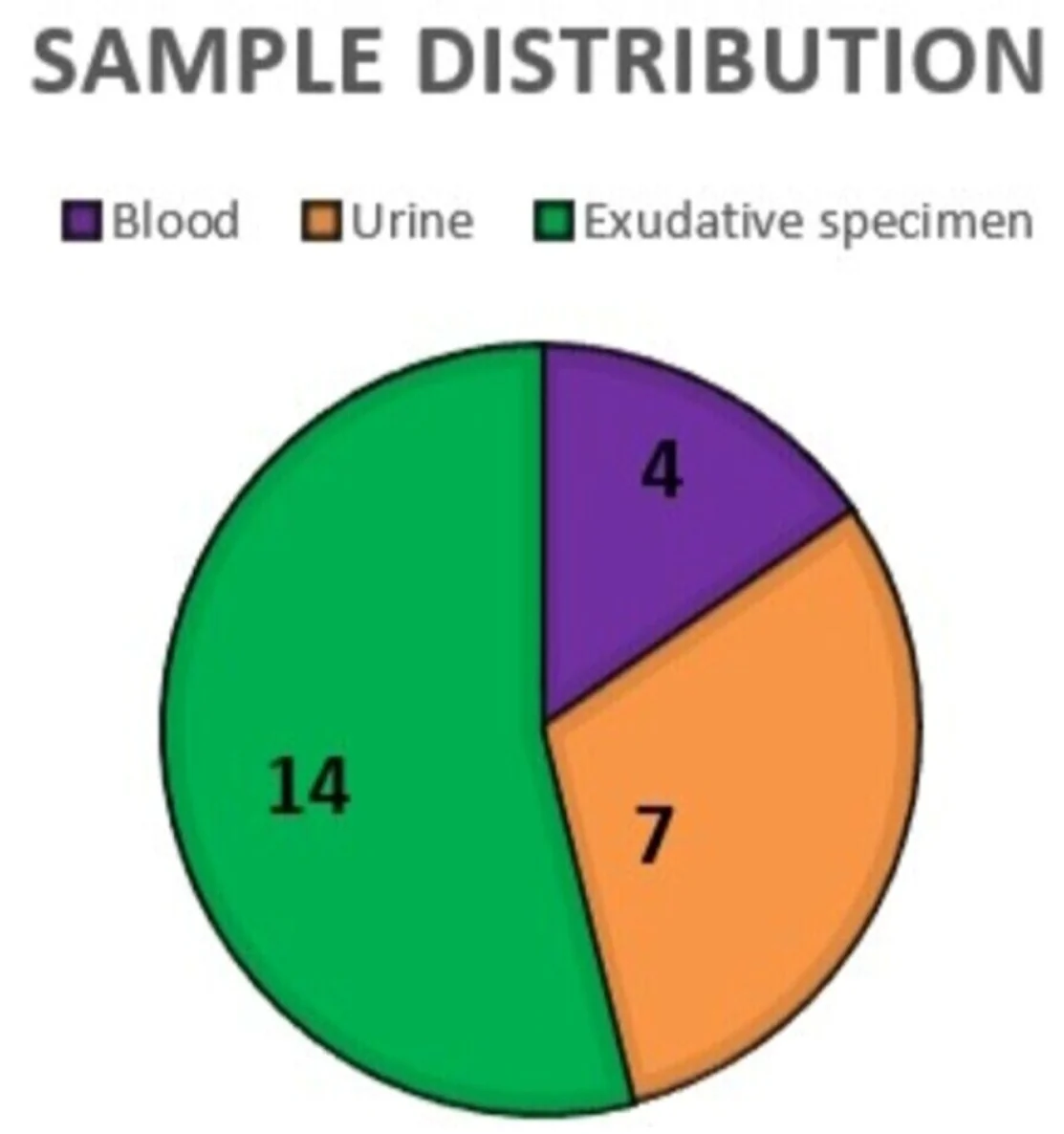
Source of the LZRE isolates.

**Figure 3:**
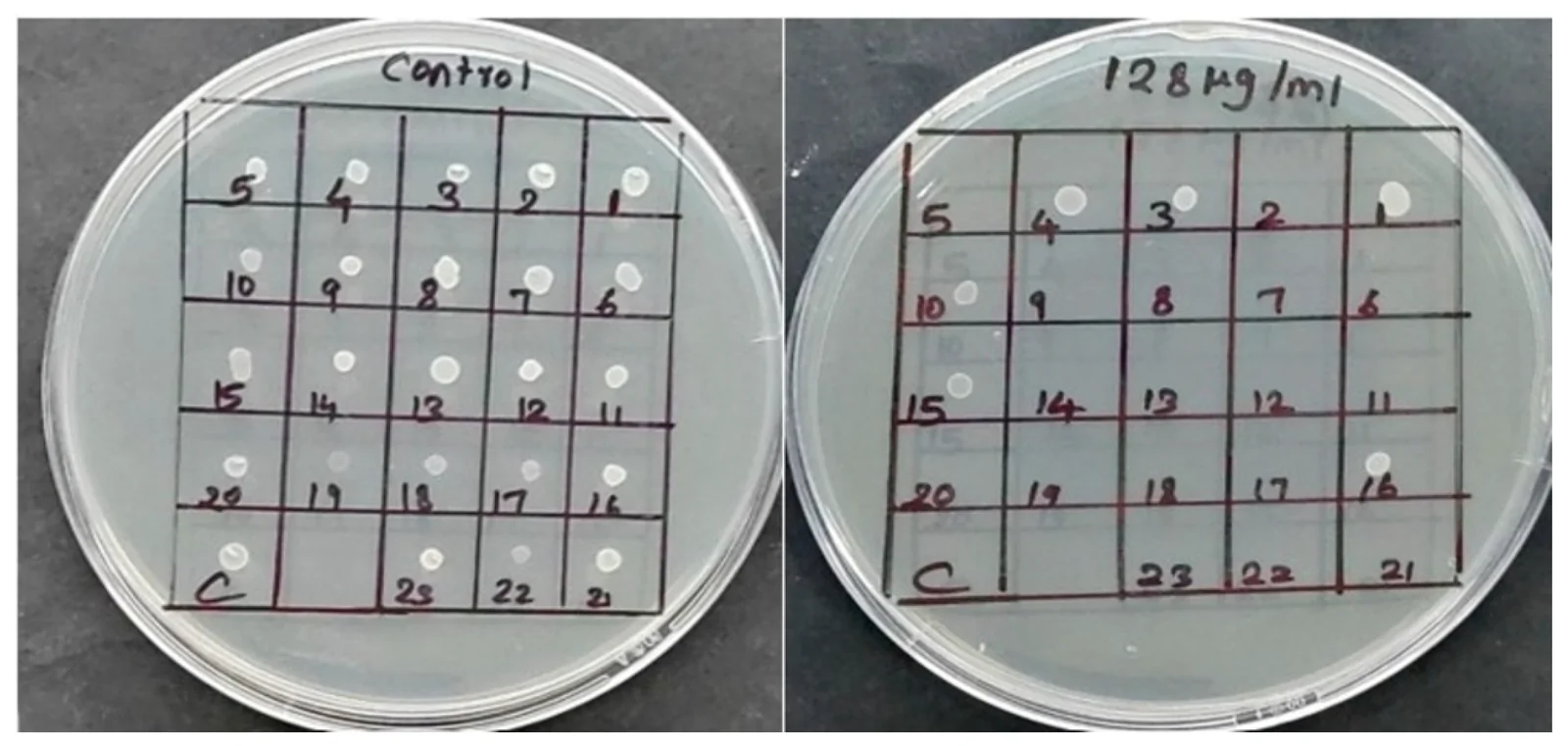
Representative MIC image of linezolid-resistant *Enterococcus* isolates.

Out of 266 isolates, linezolid resistance was detected in 25 (9.4%) isolates with MIC (≥ 8 µg/mL). Of these 25 isolates, 22 (88%) were *E. faecium*, *E. faecalis*—2 (8%), and *E. gallinarum*—1 (4%). Among them, sixteen (64%) belonged to male patients and nine (36%) to female patients. The distribution of linezolid-resistant *Enterococcus* isolates by clinical source is illustrated in Figure 2. Furthermore, the Minimum Inhibitory Concentrations (MIC) range of these isolates is listed in Table 1, with a representative MIC image depicted in Figure 3.

### 2.3. Molecular Analysis

**Figure 4:**
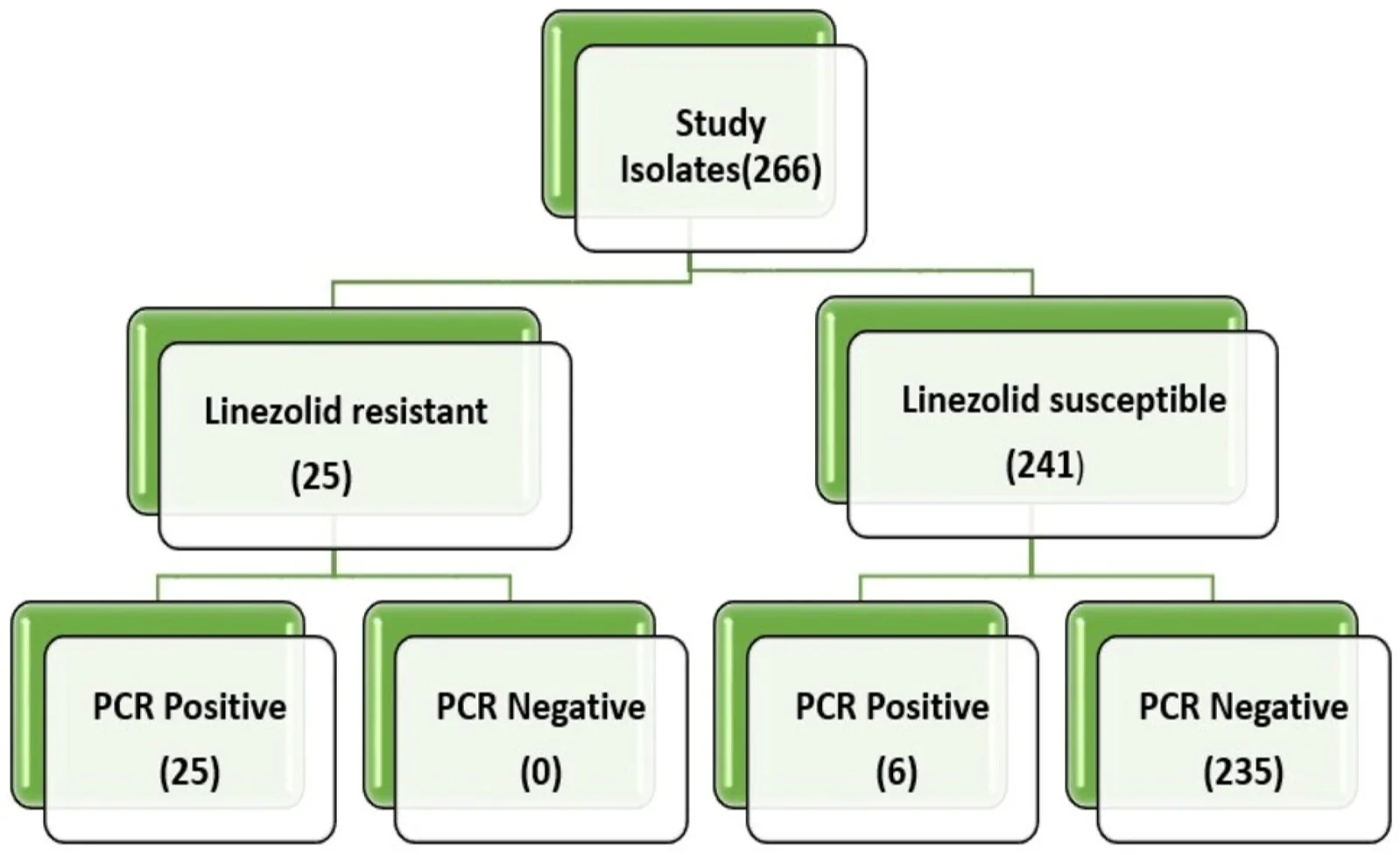
Flowchart depicting the distribution of Linezolid Resistance and PCR Detection among *Enterococcus* Isolates.

**Figure 5:**
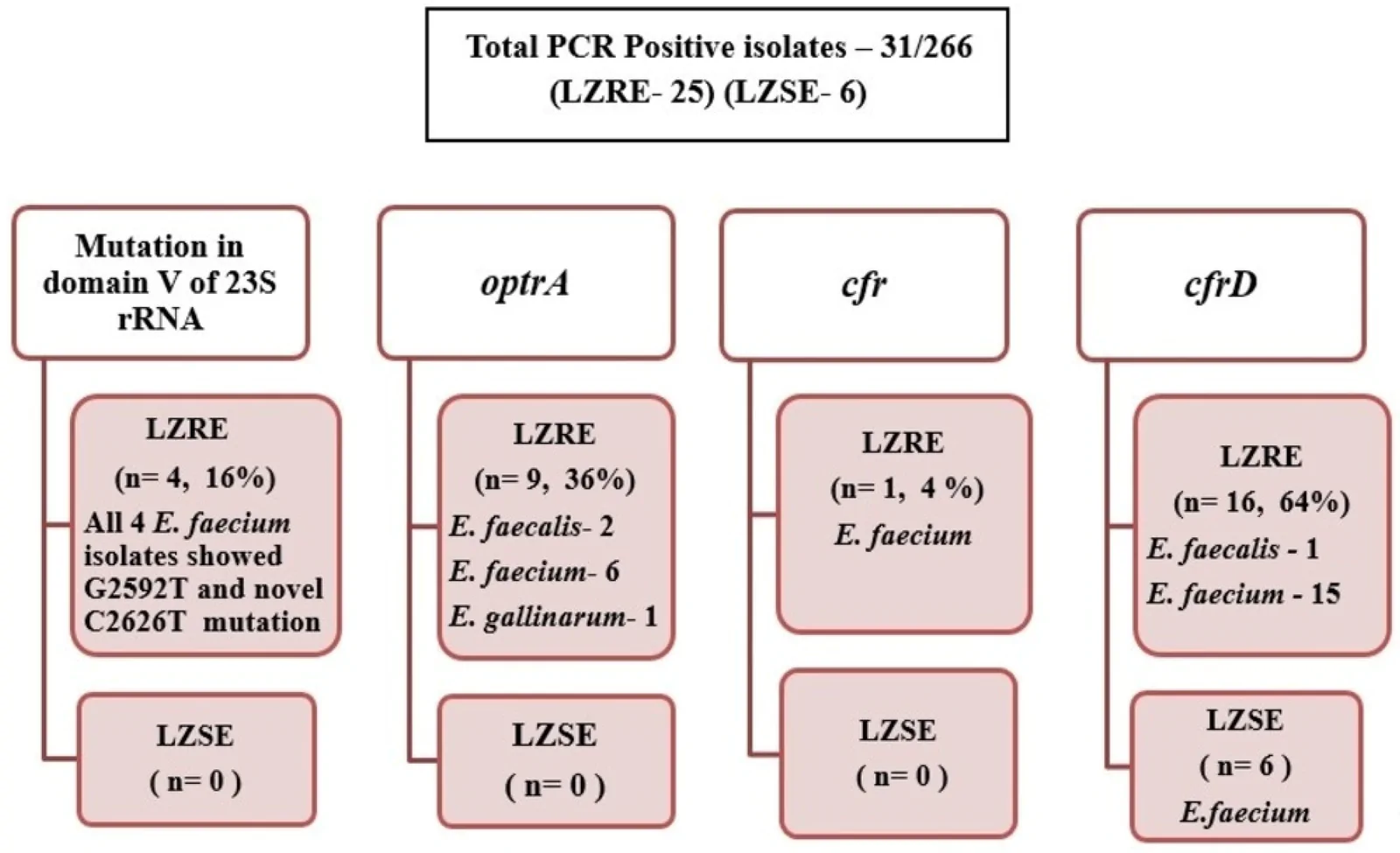
Molecular basis of linezolid resistance in clinical isolates of *Enterococcus* species. Footnote: LZRE—Linezolid-Resistant *Enterococcus*, LZSE—Linezolid Sensitive *Enterococcus*.

**Figure 6:**
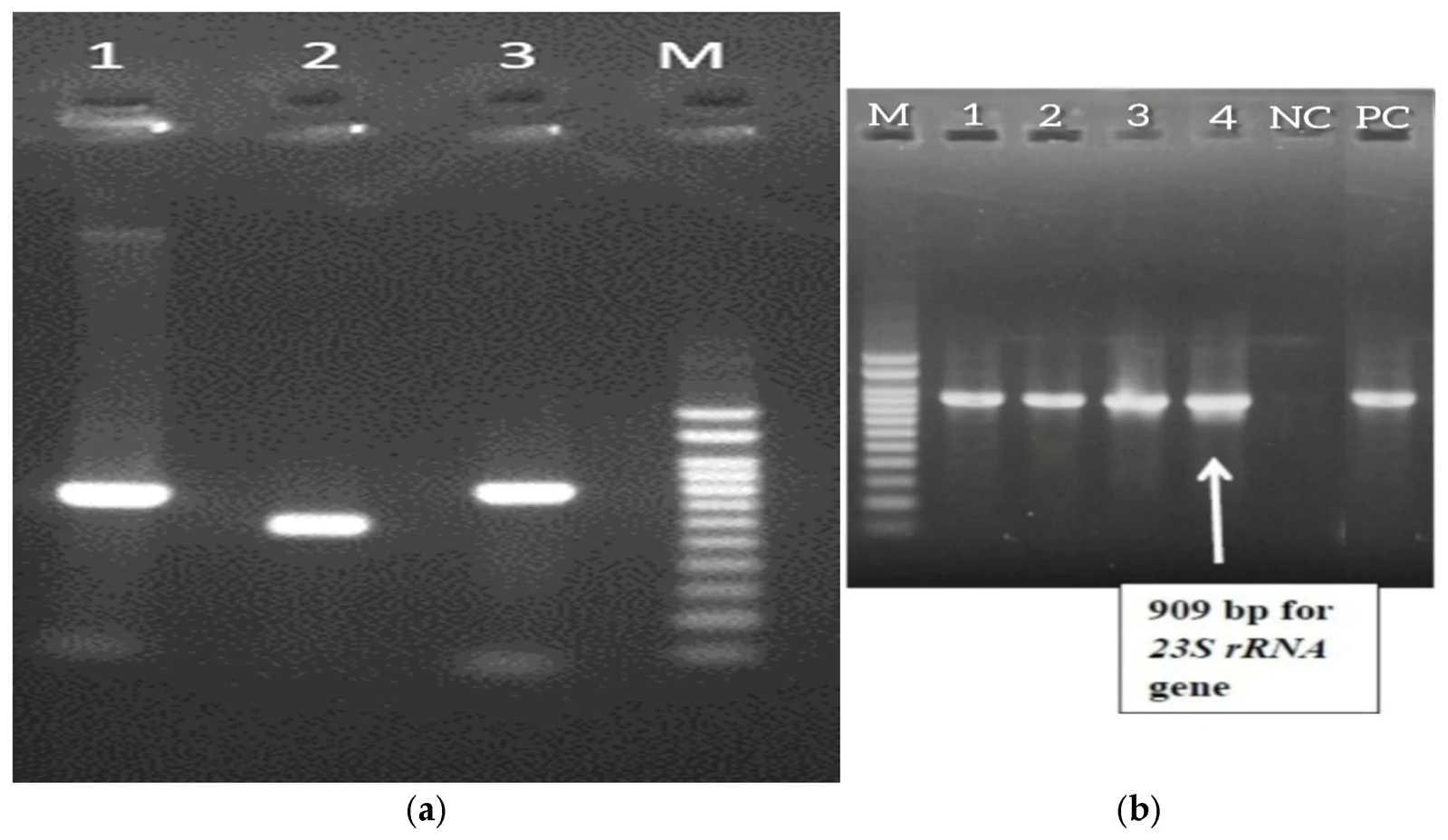
(**a**) Agarose gel electrophoresis of PCR products corresponding to Linezolid-resistant genes *cfr*, *cfr(D)*, and *optrA*. Lane 1—*cfr* (746 bp); Lane 2—*cfr(D)* (595 bp); Lane 3—*optrA* (792 bp); M—DNA Marker. (**b**) Agarose gel electrophoresis of PCR products corresponding to the 23*S rRNA* gene. M—DNA Marker; Lanes 1–4—23S rRNA gene.

**Figure 7:**
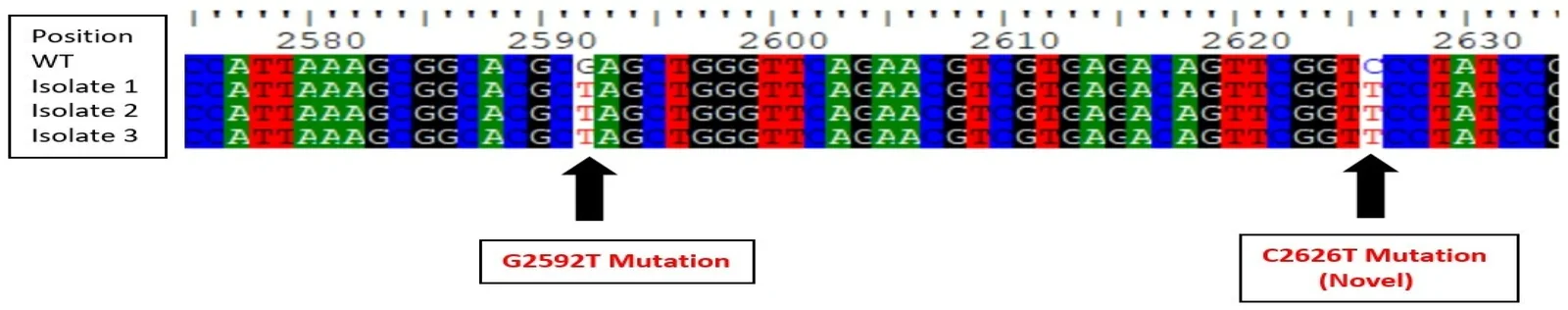
Comparative sequence analysis of the 23S rRNA gene in wild-type (WT) and clinical isolates, highlighting G2592T and a novel C2626T point mutation.

In a total of 266 isolates, 31 (11.7%) carried one or a combination of linezolid resistance genes (including *optrA*, *cfr*, *cfr(D)*, or a mutation in domain V of 23S rRNA). Molecular analysis revealed that 9 isolates carried *optrA*, 1 isolate harbored *cfr*, and 22 isolates carried the *cfr(D)* gene. Of the 22 *cfr(D)* positive isolates, six were susceptible to linezolid. The *cfr(D)* gene was present in both *E. faecium* and *E. faecalis*. Furthermore, of the twenty-five linezolid-resistant *Enterococci*, mutations in domain V of the 23S rRNA gene were observed in four *E. faecium* isolates. BLAST alignment unveiled a novel C2626T mutation, which is not previously reported in India, and a G2592T mutation (already documented in India). All four isolates displayed both the mutations with MIC > 128 (µg/mL). Two isolates harbored *optrA* with C2626T and G2592T mutations. Both *cfr(D)* and *optrA* co-occurred in three isolates. Representative PCR amplicons of *cfr*, *cfr(D)*, and *optrA* were sequenced and deposited in GenBank under accession numbers (PX531853—*cfr*, PX626519—*cfr(D)*, PX682460—*optrA*) and C2626T and G2592T mutation in 23S rRNA with the IDs PX830352, PX830353, PX830354, and PX830355. Molecular detection of linezolid resistance in *Enterococcus* species is depicted in Figure 4, Figure 5, Figure 6a,b and Figure 7. MIC stratification by species and resistance genes is illustrated in Table 2 and Table 3.

Among the 25 patients identified with LZRE, isolates were predominantly detected in individuals with major comorbidities such as Type 2 Diabetes Mellitus, systemic hypertension, chronic kidney disease (including dialysis-dependent CKD V), coronary artery disease, and other immunocompromised states. Most patients had prolonged exposure to invasive devices, including dialysis catheters, Foley catheters, arterial/central lines, and endotracheal tubes, indicating its predominance as a healthcare-associated pathogen. Notably, five patients had prior linezolid exposure, indicating that antibiotic selection pressure may have facilitated the emergence of resistance.

The 25 LZRE isolates were isolated from patients experiencing a broad spectrum of clinical conditions, highlighting the pathogen’s ability to cause both systemic and localized infections. The most frequently observed clinical conditions were sepsis and soft tissue/wound infections (including diabetic foot, cellulitis, and Fournier’s gangrene).

Demographic details of patients with linezolid resistance harboring 23S rRNA domain V mutations are listed in Table 4. Prior days of linezolid received and the level of care at the time of linezolid-resistant *Enterococcus* detection are shown in Table 5.

### 2.4. Results of Statistical Analysis

Statistical analysis was performed only for the predominant species, *E. faecalis* (128) and *E. faecium* (117). Chi-square analysis revealed a significant association between species and linezolid resistance (χ^2^ = 20.56, df = 1, *p* < 0.0001), with *E. faecium* showing higher odds of resistance than *E. faecalis* (Odds Ratio = 14.6). The association between clinically significant *Enterococcus* species and linezolid resistance is depicted in Table 6.

In addition, a significant association was observed between the presence of the mutation and high-level resistance (MIC ≥ 128; Fisher’s exact test, *p* = 0.0012). Specifically, high-level resistance was predominantly observed in mutated strains (100%, 4/4), whereas it was rarely observed in other resistance determinants (2/21). Fisher’s exact test was performed due to the small sample size and low expected frequencies within the comparison groups. Fisher’s exact test analysis is depicted in Table 7.

## 3. Discussion

*Enterococcus* species are remarkably resilient and can endure in adverse environmental conditions, which makes them well-suited for hospital settings [18]. Vancomycin-resistant *Enterococci* were categorized as a high-priority pathogen on the World Health Organization’s (WHO) 2024 worldwide list of significant antibiotic-resistant microorganisms [19]. In treating infections caused by vancomycin-resistant *Enterococci*, linezolid serves as a last effective option. Alarmingly, the emergence and dissemination of linezolid resistance among *Enterococci* has sharply increased in India and across the globe in recent times [20].

In this study, eight distinct *Enterococcus* species were identified among 266 isolates. Among these, *E. faecalis* (48.1%) was the most prevalent, followed by *E. faecium* (44.0%). This distribution aligns with findings from other studies [21,22] where *E. faecalis* is typically predominant than other species.

In our study, (25/266) 9.4% of clinical *Enterococcus* isolates exhibited resistance to linezolid (MIC ≥ 8 µg/mL). Whereas, other studies from India reported 16% [23] in clinical *Enterococcus* isolates and 3.06% [24] among urinary *Enterococcus* isolates. Linezolid resistance was observed in *E. faecium* (22/25, 88%), *E. faecalis* (2/25, 8%), and *E. gallinarum* (1/25, 4%) in this study. Of these, *E. faecium* is predominant in exhibiting resistance to linezolid. Amongst 25 LZRE, 8 clinical isolates were from the ICU, while the remaining 17 were from non-ICU wards. The majority of isolates were obtained from exudative specimens, followed by urine and blood. Most of the linezolid resistance *Enterococcus* species also exhibited resistance to ampicillin (24/25) and vancomycin (13/25).

Linezolid resistance in *Enterococcus* is facilitated by mutations in domain V of 23S rRNA, acquiring *cfr*, *cfr(D)*, and *optrA* resistance genes. Based on the review of the literature, the most common mutations encompass G2576T, G2447U, and G2504A [25]. Nevertheless, this study did not observe any of the aforementioned mutations. To the best of our knowledge, this study reports a novel C2626T mutation in domain V of 23S rRNA in clinical isolates of *E. faecium*. In addition to that, a common G2592T mutation is also observed in this study. Similarly, a South Indian study also reported the G2592T mutation [16]. Previously, mutations in 23S rRNA of domain V were thought to be the main mechanism of linezolid resistance among clinical isolates. Recent research [25,26,27], including this study, has highlighted evolving mechanisms of resistance in hospital settings.

The *optrA* gene was first identified in *E. faecalis* from human sources in China in 2015 [28]. Following this, a nationwide surveillance study across China found that 2% of enterococcal isolates possessed the *optrA* gene. Additionally, research has shown that *optrA*-positive *Enterococci* are more prevalent in animals and are increasingly being reported in human infections [29]. For example, a study from Italy reported *optrA* carrying linezolid-resistant *E. gallinarum* of swine origin [30]. In our study, (9/25) 36% of linezolid-resistant *Enterococcus* of clinical isolates (*E. faecalis*, *E. faecium*, and *E. gallinarum*) possessed the *optrA* gene. A study from India conducted whole genome sequencing and reported an *Enterococcus faecium* strain carrying the *optrA* gene with the G2592T mutation [16]. Whereas, in our study results, *optrA* harboring linezolid-resistant *E. faecium* with both C2626T and G2592T mutation was observed.

To the best of our knowledge, this study reports *cfr*- and *cfr(D)*-mediated linezolid resistance in *Enterococcus* species from India. Although *cfr* and *cfr(D)* genes have previously been reported in other countries, reports from India are scarce. In addition, a novel C2626T mutation was identified among the resistant isolates. A study from Thailand documented transferable plasmid-mediated resistance conferred by the *cfr* gene in a clinical strain of *Enterococcus faecalis* [31].

The *cfr(D)* gene was initially found in a clinical isolate of *E. faecium* from France in 2015 [12] and later in a blood isolate from an Australian patient in 2019 [32]. The presence of *cfr(D*) in a clinical isolate of *E. faecalis* from Spain in 2020 [12]. In our study, 22 clinical isolates carried the *cfr(D)* gene. Among them, sixteen were resistant to linezolid, while the other six were phenotypically susceptible to linezolid. This may be due to incomplete gene expression. Both *cfr(D)* and *optrA* coexisted in three clinical isolates of linezolid-resistant *Enterococcus* (*E. faecalis*-1, *E. faecium*-2). In our study, *cfr(D)* is the predominant mechanism of Linezolid-resistant *Enterococcus*. The coexistence of clinical conditions with transferable resistance genes and chromosomal mutations may contribute to the persistence of resistant strains in healthcare environments. This underscores the need for continued surveillance, prudent antimicrobial use, and effective infection control practices.

## 4. Materials and Methods

### 4.1. Ethics Approval of Research

The Institutional Ethics Committee of Sri Ramachandra Institute of Higher Education and Research approved the study protocol (REF: IEC-N1/23/AUG/88/43).

### 4.2. Study Design and Setting

This in vitro, laboratory-based, prospective, cross-sectional study was carried out in a 1600-bedded teaching hospital in Chennai, South India. A total of 266 clinically significant, consecutive, non-repetitive isolates collected between 2023 and 2024 were analyzed in this study. Sources of the isolates included urine (*n* = 98), blood (*n* = 19), and exudative specimens (*n* = 149). The significance of the isolates was determined by correlating them with clinical history, detecting intracellular organisms in Gram staining, and observing significant growth on culture media. Species were identified through conventional biochemical tests and automated methods, including matrix-assisted laser desorption ionization-time-of-flight mass spectrometry (MALDI-TOF MS) from bioMérieux Marcy l’Etoile, France.

### 4.3. Antimicrobial Susceptibility Testing

Antimicrobial susceptibility was tested for various antimicrobial agents, including ampicillin (10 µg), vancomycin (30 µg), and linezolid (30 µg), using the Kirby-Bauer disc diffusion method and interpreted as per Clinical and Laboratory Standards Institute 2025 guidelines. All urinary isolates were tested for ciprofloxacin (5 µg) and nitrofurantoin (300 µg). Erythromycin (15 µg) only for non-urinary isolates.

### 4.4. Minimum Inhibitory Concentration

The agar dilution technique was used to ascertain the MIC for linezolid. Mueller-Hinton agar plates were formulated from a stock solution using 12 distinct concentrations ranging from 0.125 µg/mL to 128 µg/mL. Three to four colonies of *Enterococcus* isolates were transferred into peptone broth, adjusted to a 0.5 McFarland standard, and incubated for 20 min. Afterward, the inoculum was placed onto gridded Mueller-Hinton agar plates using a sterile loop in the specified grids. *Enterococcus faecalis* ATCC 29212 was used as a quality control strain. The plates were then incubated at 37 °C, and results were recorded after 24 h. If the MIC was ≥8 µg/mL, isolates were classified as phenotypically resistant to linezolid, as per the Clinical and Laboratory Standards Institute 2025 guidelines.

### 4.5. Molecular Methods

#### 4.5.1. DNA Extraction

Clinical *Enterococcus* colonies were inoculated into Luria-Bertani broth (HiMedia Laboratories, Mumbai, Maharashtra, India) and incubated at 37 °C for 16 h. Following this, the culture underwent centrifugation at 10,000 rpm for 10 min. The resulting pellet was reconstituted in 400 μL of sterile water and boiled at 100 °C for 10 min. After cooling, the suspension underwent a second centrifugation at a speed of 10,000 rpm for a span of 10 min. The resulting supernatant was utilized as the template DNA in the following Polymerase Chain Reaction (PCR) analysis [33].

#### 4.5.2. Polymerase Chain Reaction:

The presence of genes conferring linezolid resistance (*optrA*, *cfr*, *cfr(D)*, and a mutation in domain V of 23S rRNA) was determined by the PCR technique. For each PCR reaction, a total volume of 10 µL was used, containing 5 µL of master mix (Takara, New Delhi, India), 2 µL of sterile Milli-Q water, 0.5 µL of each primer, and 2 µL of template DNA. Amplified products were resolved on a 2% agarose gel with ethidium bromide for visualization [33]. PCR was performed with previously characterized positive control strains and sterile Milli-Q water as a negative control. Primer specifications are listed in Table 8.

#### 4.5.3. DNA Sequencing and Analysis

DNA sequencing was performed using the Sanger sequencing method on an ABI 3730XL sequencer (Applied Biosystems, Foster City, CA, USA) with the ABI PRISM BigDye Terminator kit. The resulting nucleotide sequences were analyzed and aligned using BioEdit software version 7.0.5.3. Sequence similarity searches were conducted using the Basic Local Alignment Search Tool (BLAST) +2.17.0 available through the National Center for Biotechnology Information (NCBI) database. The finalized sequences were submitted to GenBank, and accession numbers were obtained.

#### 4.5.4. Statistical Analysis

All statistical analyses were performed using GraphPad Prism version 11.0.2, and *p*-values < 0.05 were considered statistically significant.

## 5. Conclusions

The findings of this study highlight the concerning evolution of linezolid resistance among clinical *Enterococcus* species, which threatens the effectiveness of this last-resort antibiotic. The identification of a novel C2626T mutation with high MIC values, in addition to the significant prevalence of resistance genes such as *cfr and cfr(D)* and their concerning coexistence with *optrA*, underscores a shift towards multifaceted resistance mechanisms. Notably, *cfr(D)* was the predominant genetic determinant identified in this study. Moreover, the detection of *cfr(D)* in isolates lacking phenotypic resistance emphasizes the importance of molecular surveillance, as these isolates may facilitate silent dissemination and rapid acquisition of resistance under linezolid selective pressure. To mitigate the spread of these multidrug-resistant isolates, the implementation of strengthened infection control practices, rational antimicrobial use, and early detection is an essential step for effective containment and preservation of available therapeutic options in healthcare settings.

## Figures and Tables

**Table 1:** Minimum Inhibitory Concentration of linezolid against resistant *Enterococcus* isolates.

**MIC Range (µg/mL)**	0.125	0.25	0.5	1	2	4	8	16	32	64	128	>128
**No. of isolates**	0	1	5	71	164	0	1	8	3	7	0	6

[Results interpreted as per CLSI guidelines 2025. Sensitive—≤2 (µg/mL), Intermediate—4 (µg/mL), Resistant—≥8 (µg/mL)]. The MIC_50_ and MIC_90_ values for all 266 *Enterococcus* isolates were 2 µg/mL and 2 µg/mL, respectively. Among linezolid-resistant isolates (*n* = 25), MIC_50_ and MIC_90_ were 64 µg/mL and >128 µg/mL, respectively.

**Table 2:** MIC stratification by species.

Species	0.125	0.25	0.5	1	2	4	8	16	32	64	128	>128	Total
*E. faecalis*	0	0	0	42	84	0	0	1	0	0	0	1	128
*E. faecium*	0	0	0	24	71	0	0	7	3	7	0	5	117
*E. gallinarum*	0	0	3	2	1	0	1	0	0	0	0	0	7
*E. casseliflavus*	0	1	0	0	0	0	0	0	0	0	0	0	1
*E. raffinosus*	0	0	1	0	1	0	0	0	0	0	0	0	2
*E. avium*	0	0	0	3	3	0	0	0	0	0	0	0	6
*E. durans*	0	0	1	0	0	0	0	0	0	0	0	0	1
*E. hirae*	0	0	0	0	4	0	0	0	0	0	0	0	4
Total	0	1	5	71	164	0	1	8	3	7	0	6	266

**Table 3:** MIC stratification according to resistant genes.

MIC Range	*cfr*	*cfr(D)*	*OptrA*	Mutation
0.125	0	0	0	0
0.25	0	0	0	0
0.5	0	0	0	0
1	0	1	0	0
2	0	5	0	0
4	0	0	0	0
8	0	0	1	0
16	0	6	3	0
32	0	3	0	0
64	0	7	2	0
128	0	0	0	0
>128	1	0	3	4

Footnote: [Results interpreted as per CLSI guidelines 2025. Linezolid Sensitive—≤2 (µg/mL), Linezolid Intermediate—4 (µg/mL), Linezolid-Resistant—≥8 (µg/mL)].

**Table 4:** Summarizes the demographic characteristics of patients with linezolid-resistant isolates carrying 23S rRNA domain V mutation.

Characteristics	Patient 1	Patient 2	Patient 3	Patient 4
Isolate number	GE4273	GE5439	GE7556	GE492
Age/Sex	54 M	63 M	50 F	69 M
Hospital location	Ward	Ward	Ward	Ward
*Enterococcus* species	*E. faecium*	*E. faecium*	*E. faecium*	*E. faecium*
Admitting specialty	Plastic Surgery	General Surgery	General Surgery	Dermatology and venereology
Final diagnosis	50% total body surface area electrical burns of the bilateral upper limb, left lower limb, and back	UGI Bleed (Resolving); Septic shock (Resolved)- UTI, Grade IV Bed sore, status post debridement of bedsore Grade IV	Diabetic foot ulcer, Status post ray amputation of the right fourth and fifth toes	Left leg cellulitis S/P wound debridement and fasciotomy, Bilateral lower limb DVT
Co-morbid conditions	nil	Type 2 diabetes mellitus, bipolar disorder	Type 2 diabetes mellitus	Type 2 diabetes mellitus
Days in hospital	61	45	41	39
Surgical procedures	Wound debridement, escharotomy, fasciotomy	Wound debridement	Ray amputation	Wound debridement and fasciotomy
Antimicrobials used prior to detection of linezolid resistance	Linezolid	-	-	Linezolid
Outcome	Recovered	Death	Recovered	Recovered
Source specimen	Tissue	Pus cls	Tissue	Pus cls
MIC (µg/mL)	>128	>128	>128	>128
23S rRNA mutations in domain V	C2626T and G2592T	C2626T and G2592T	C2626T and G2592T	C2626T and G2592T
*cfr*, *cfr(D)*	Nil	Nil	Nil	Nil
*optrA*	Nil	Nil	*optrA*	*optrA*

**Table 5:** Clinical and molecular profiles of linezolid-resistant *Enterococcus* isolates from patients with prior Linezolid exposure.

S.NO	Species	Sample Source	Prior Days of Linezolid Received	Level of Care During Linezolid Exposure	Level of Care After Linezolid Detection	Gene	Clinical Outcome
1	*E. faecium*	Tissue	31 days	ICU	Ward	C2626T and G2592T MIC ≥128 (µg/mL)	Recovered
2	*E. gallinarum*	Blood cls	8 days	Ward	ICU	*optrA* MIC 8 (µg/mL)	Death
3	*E. faecium*	Pus cls	8 days	ICU	Ward	C2626T and G2592T MIC ≥128 (µg/mL)	Death
4	*E. faecium*	Tissue cls	5 days	Ward	Ward	*cfr* MIC ≥128 (µg/mL)	Recovered
5	*E. faecium*	Pus cls	12 days	ICU	Ward	C2626T and G2592T MIC ≥128 (µg/mL)	Recovered

**Table 6:** Chi-square analysis of linezolid resistance among *E. faecalis* and *E. faecium*.

Species	LZSE	LZRE	Total	χ^2^, df	*p*-Value
*E. faecalis*	126	2	128	20.56, df = 1	<0.0001
*E. faecium*	95	22	117		
Total	221	24	245		

Footnote: This study identified 25 linezolid-resistant *Enterococcus* (LZRE) isolates, including 22—*E. faecium*, 2—*E. faecalis*, and 1—*E. gallinarum* isolate. Table 6 presents the 2 × 2 contingency analysis comparing the two predominant species, *E. faecalis* and *E. faecium* (Total *n* = 24). The *E. gallinarum* isolate (*n*=1) was not included in the chi-square analysis. (*p*-value < 0.0001 = statistically significant).

**Table 7:** Contingency table showing the association between mutation status and high-level linezolid resistance (Fisher’s exact test).

Genotype	High Level Resistance ≥ 128 µg/mL	Resistance ≤ 128 µg/mL	Total	*p* Value
Mutation	4	0	4	*p* = 0.0012
other gene	2	19	21	
Total	6	19	25	

**Table 8:** PCR primers and cycling conditions for linezolid resistance genes.

Gene	Primer	PCR Conditions	Base Pair	Ref.
23S rRNA	F: GTAACGATTTGGGCACTGTCG R: CGATTAGTATTGGTCCGCTC	Initial denaturation: 94 °C for 5 min, followed by 30 cycles of Denaturation: 94 °C for 30 s, Annealing: 55 °C for 30 s Extension: 72 °C for 30 s Final Extension: 72 °C for 5 min	909	[33]
*optrA*	F: CAGGTGGTCAGCGAACTAAGA R: AGCCAAGAGCAGTTCTGACC	Initial denaturation: 94 °C for 5 min followed by 30 cycles of, Denaturation: 94 °C for 30 s, Annealing: 56 °C for 30 s, Extension: 72 °C for 30 s, Final Extension: 72 °C for 5 min	792	[34]
*Cfr*	F: TGAAGTATAAAGCAGGTTGGGAT R: ACCATATAATTGACCACAAGCAGC	Initial denaturation: 94 °C for 2 min, Denaturation: 94 °C for 10 s, Annealing: 55 °C for 30 s, Final extension: 7 min at 72 °C	746	[35]
*cfr(D)*	F: CAGGTGGTCAGCGAACTAAGA R: AGCCAAGAGCAGTTCTGACC	Initial denaturation: 94 °C for 7 min, followed by 30 cycles of Annealing: 60 °C for 1 min, Extension: 72 °C for 1min, Final extension: 72 °C for 10 min.	595	[36]

## Data Availability

The data presented in this study are openly available in NCBI at https://www.ncbi.nlm.nih.gov/nuccore/PX531853 (accessed on 26 May 2026), reference number PX531853, PX626519, PX682460, PX830352, PX830353, PX830354.
